# Wireless Stimulation of Antennal Muscles in Freely Flying Hawkmoths Leads to Flight Path Changes

**DOI:** 10.1371/journal.pone.0052725

**Published:** 2012-12-26

**Authors:** Armin J. Hinterwirth, Billie Medina, Jacob Lockey, David Otten, Joel Voldman, Jeffrey H. Lang, John G. Hildebrand, Thomas L. Daniel

**Affiliations:** 1 Department of Biology, University of Washington, Seattle, Washington, United States of America; 2 Department of Electrical Engineering and Computer Science, Massachusetts Institute of Technology, Cambridge, Massachusetts, United States of America; 3 Department of Neuroscience, University of Arizona, Tucson, Arizona, United States of America; University of California Los Angeles, United States of America

## Abstract

Insect antennae are sensory organs involved in a variety of behaviors, sensing many different stimulus modalities. As mechanosensors, they are crucial for flight control in the hawkmoth *Manduca* sexta. One of their roles is to mediate compensatory reflexes of the abdomen in response to rotations of the body in the pitch axis. Abdominal motions, in turn, are a component of the steering mechanism for flying insects. Using a radio controlled, programmable, miniature stimulator, we show that ultra-low-current electrical stimulation of antennal muscles in freely-flying hawkmoths leads to repeatable, transient changes in the animals' pitch angle, as well as less predictable changes in flight speed and flight altitude. We postulate that by deflecting the antennae we indirectly stimulate mechanoreceptors at the base, which drive compensatory reflexes leading to changes in pitch attitude.

## Introduction

All insects possess antennae [1]. While in many species these appendages primarily function as olfactory and tactile organs, in flying insects antennae also subsume a mechanosensory role that is crucial for flight control. For example, antennae have been associated with air flow sensing, through behavioral evidence indicating that intact antennae are necessary for flight speed regulation in bees, flies, locusts, dragonflies, and butterflies [2]–[7]. More recently, antennal mechanoreceptors have also been shown to be important for inertial sensing providing sensory feedback that counteracts rapid perturbations of an insect's body orientation. Sane et al. [8] discovered that flight performance depends on the presence of the antennal flagellum. The latter acts as a proof mass for mechanosensors, most likely Johnston's organ, located at the base of each antenna. The sensory neurons are neurophysiologically tuned to the mechanical signals expected from inertial strain signals occurring during rotations of the body [8].

Specifically, the mechanosensors at the base of the antennae mediate an abdominal flexion response to rotations that can be employed in steering [9]. As with the free-flight experiments conducted by Sane *et al.*, cutting the flagella off their bases leads to a loss of function; gluing them back rescues the response. In addition to being driven by antennal mechanosensors, abdominal flexion is elicited by visual stimuli. In fact, the visual system, by measuring rotational optic flow, leads to even stronger abdominal responses, which are almost anti-phase to the ones elicited mechanically.

As a further complication, antennae are not merely passively moved by wind or inertial forces that happen during body turns. In *Manduca sexta*, there are two sets of small muscles that control antennal posture: 1.) extrinsic muscles that attach in the tentorium within the head capsule and insert onto the proximal scape; and 2.) intrinsic muscles within the scape that insert onto the pedicel [10]. For flight, these muscle groups bring antennae from a recessed rest position into a forward-facing flight position permitting wind and rotation sensing. When in flight mode, multiple feedback circuits act on the muscles. One keeps the inter-antennal angle constant and independent of air speed [11], which can provide a means of regulating flight speed [6]–[7].

Thus, the behavior of a flying moth is the complex result of combining information from multiple sensors acting over many neural circuits to affect multiple actuators. To gain a better understanding of the role of a subsystem to this complex cascade, namely the antennal mechanosensors, perturbation studies can lead to important insights [12]. We therefore asked whether in-flight stimulation of these sensors can lead to predictable changes in a moth's flight trajectory.

Due to the challenges posed by experiments on a freely flying animal, we decided to use extrinsic antennal muscle stimulation as a proxy for stimulation of antennal mechanosensors, driving antennal motions via stimulation of their supporting muscles. We first show that stimulation of extrinsic muscles in resting animals leads to transient deflections of the antennae. Such deflection necessarily leads to changes in strain at the antennal base, stimulating local mechanosensors. When using the same stimulation paradigm in freely flying moths by employing a telemetrically controlled ultra-low mass current stimulus board, we find that transient changes in the moth's pitch angle are the most reliable response. Observed changes in the flight path elicited by antennal stimulation is consistent with the earlier finding that mechanical rotations in tethered moths lead to an abdominal flexion that would lead to changes in the animals' pitch orientation.

## Materials and Methods

### Animals

*Manduca sexta* were reared in the Department of Biology at the University of Washington, Seattle. All experiments used male moths with no apparent defects in wings or eyes 3–5 days after eclosion. Animals were usually chilled in a refridgerator at ∼10°C for about 10 minutes prior to preparing them for an experiment.

### Antennal stimulation and deflection measurements

**Figure 1:**
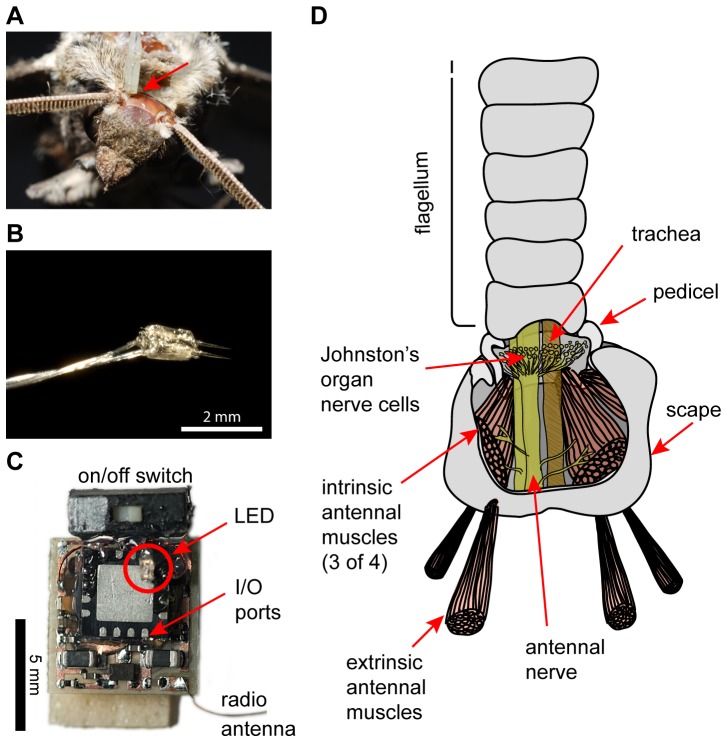
Overview of how antennal stimulation in free-flying animals was achieved. (**A**) Top view of a moth's head, with one electrode pair placed (indicated by red arrow), but not yet waxed down, to target extrinsic antennal muscles. The other electrode pair has not yet been placed. (**B**) Photograph of a typical pair of tungsten electrodes used for electrical stimulation of extrinsic muscles. (**C**) Photograph of the “RadioFlyer” microcircuit that is mounted ventrally on a moth to provide telemetrically triggered electric muscle stimulation. (**D**) Simplified schematic (redrawn and modified from [10]) showing the two muscle groups involved in positioning a moth's antennae. Extrinsic muscles, which move the whole antenna with respect to the head, were targeted for the experiments presented here.

Electrical stimuli were delivered to extrinsic antennal muscles by inserting fine stimulation electrodes to a depth of ∼1 mm, in a dorso-medial location near each antennal rim on the head capsule (Fig. 1). All stimuli consisted of 2.8–3 V square-pulse signals of 50% duty cycle and varying frequencies. They were delivered by a miniature stimulator board based on a PIC16F688 microprocessor (Microchip Technology Inc., Chandler, AZ). A transmitter that communicated with the stimulator board wirelessly was connected to a computer running custom-written software (implemented in Java) to cycle through the different stimulus parameters, and stimulate the antennal muscles every 5 seconds.

A high-speed video camera (Phantom Miro 4; Vision Research, NJ) filmed the moth from above (at 250 fps) and the tip and base coordinates of the stimulated antenna were extracted with custom MATLAB digitizing software (DLTdataviewer [13]) to calculate deflection angles for each frame.

Only antennal motions were elicited by the electrical stimuli when electrodes were placed correctly. We only kept trials in which antennal motion mainly occurred in a plane extending backwards and slightly upwards because other directions tended to also show responses beyond just antennal movement (such as leg twitching). Due to this directionality we assume that our target muscles are mainly the anterior or posterior levator muscles (ALM, PLM in [10]). Imperfectly placed electrodes also elicited other behaviors, such as extension of legs, or movement of the contralateral antenna, suggesting that stimulus delivery was less isolated than is possible when placed into intrinsic muscles of the scape. Animals which showed such stimulus leakage, or antennal deflection in a direction other than backwards, were discarded after a tethered pre-flight test.

### Stimulation of extrinsic muscles in free flight

Stimulus electrode placement for free-flight experiments was the same as above. The stimulator board, which is small and light enough to be carried by a moth (6.8×10.2×5.1 mm; weight about 420 mg), was mounted on the ventral side of the animal, just posterior to the junction between thorax and abdomen. Before each experiment, the stimulus frequency was titrated to elicit backwards antennal deflection, and no other visible behavioral responses. We were unable to keep the electrodes and chip in place for prolonged periods of time, so all experiments were performed on the day of electrode placement. For all trials reported here, the stimulation paradigm consisted of 200 ms long trains of square pulses at 2.8–3 V, 250 Hz and a duty cycle of 50%.

### Videography and analysis of 3D trajectories

We filmed moths flying in a custom-built wind tunnel (air speed set to ca. 1 m/s) with three synchronized high-speed video cameras (Basler Pilot GigE, Basler Vision Technologies, Ahrensburg, Germany) operated at 150 fps. All cameras were synchronized with a pulse signal generated in the StreamPix 4 recording software used for visualization (NorPix, Inc. Montreal, Canada), and output through a National Instruments DAQ board (DAQ SPECS, NI, Austin, TX). A custom-built trigger circuit, based on an Arduino board (Arduino Duemilanove; SparkFun Electronics, Boulder, CO), elicited stimulation of the moth, as well as triggered the cameras, either when the moth blocked two crossed laser beams (632 nm, red low-power laser diodes), or when a push-button switch was pressed.

The three-dimensional coordinates of stimulated moths were reconstructed using customized routines (DLTdataviewer; [13]) in MATLAB (MathWorks, Natick, MA, USA). Once the 3D trajectory was extracted from a video, the moth's body angle (tip of abdomen to rostrum of the head) was calculated for each frame. The pitch angle is defined as the angle between the body vector and its projected component onto the x/y plane. The yaw orientation is the angle between the body vector's x/y component and the x-axis, which corresponds to the longitudinal axis of the wind tunnel. Altitude is the height (z-coordinate) of the center of the body vector, and speed is the absolute value of the numeric derivative of the center's position.

To quantify responses to stimulation, a 200 ms period before (T_pre_), and 200 ms after stimulus onset (T_post_) was compared: 1.) for the pitch and yaw response (ΔTheta), we calculated the difference between the mean angle during T_pre_ and the maximum or minimum deflection during T_post_. 2.) To quantify altitude and speed changes, we fit linear regressions to the data in the *pre* and *post* periods and calculated the difference in the slopes to get a mean difference in the climb rate (cm/s) or change in speed (m/s^2^), respectively.

## Results

### Electrical stimulation of intrinsic antennal muscles elicits antennal motion

**Figure 2:**
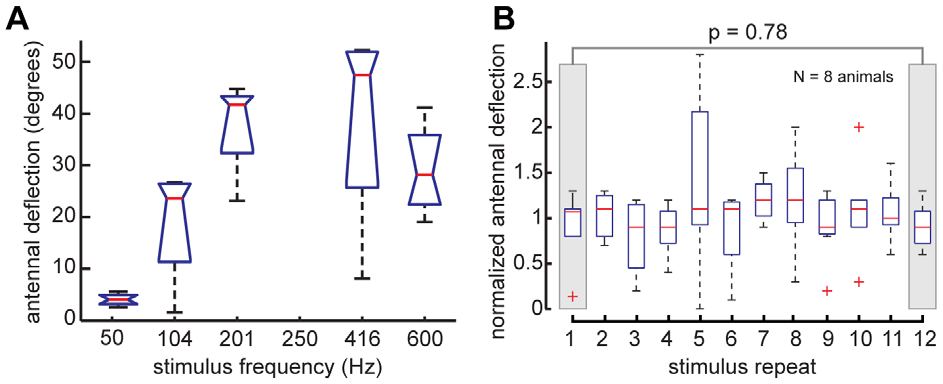
Antennal motion evoked by electrical stimulation of extrinisic antennal muscles in tethered moths. (**A**) Changing the stimulus frequency of a 3 V, 50% duty-cycle pulse train of 200 ms duration leads to changes in antennal motion: Increasing stimulus frequency leads to an increase in antennal deflection amplitude. (**B**) Antennal deflection amplitudes of 8 animals to 12 consecutive electrical stimulation events of extrinsic muscles. For each animal, the deflection amplitudes are normalized to the mean deflection for all 12 stimulus repeats. There is no significant difference between deflection amplitudes elicited by the first compared to the last stimulus trains (gray boxes; t-test p = 0.78). Likewise, linear fits to each animal's responses show a negligible trend in any direction. The overall mean slope for all fits is 0.003/stimulus event (S.D. = 0.043).

To confirm whether electrical stimulation of antennal muscles is feasible (given the power limitations of the stimulus chip used for free-flight experiments), we implanted tungsten electrodes next to the antennal rim on the head capsule to target extrinsic antennal muscles (see Figure 1A). Using a 3 V, pulse width modulated (PWM) stimulus paradigm (pulse train duration: 200 ms), we found that stimulus trains between 100 and 400 Hz lead to robust antennal deflections (see Figure 2A, supplemental Movie S1). The exact direction of the deflection depended critically on electrode implantation and was hard to repeat in a consistent fashion. We limited our analysis, as well as free-flight experiments, to animals in which stimulation lead to a qualitatively similar backwards motion of the antennal flagellum. Motion in other directions tended to be correlated with responses other than antennal motion, such as twitching of the legs or proboscis, indicating stimulus leakage. Very high stimulation frequencies might lead to recruitment of antagonistic muscles, and therefore lead to an overall decrease in deflection amplitude (see decreased response to 600 Hz stimulus in Figure 2A). Thus, depending on electrode placement, it was possible to tune the stimulus paradigm to maximize the resultant antennal deflection.

The deflection response did not fatigue significantly when stimulus trains with the same parameters (3 V, 250 Hz pulse trains with a 50% duty cycle and 200 ms pulse train duration) were repeated every five seconds for 12 successive stimulus presentations. When such repeated stimuli were presented to 8 moths, the amplitudes of antennal deflection elicited by successive stimulation events changed by an average of 0.3% per stimulus presentation (S.D. 4.3% per stimulus; see Figure 2B). A t-test between the normalized deflections due to the first and due to the last stimulus train revealed no significant difference between the two distributions (p = 0.78).

### Antennal stimulation in freely-flying moths

**Figure 3:**
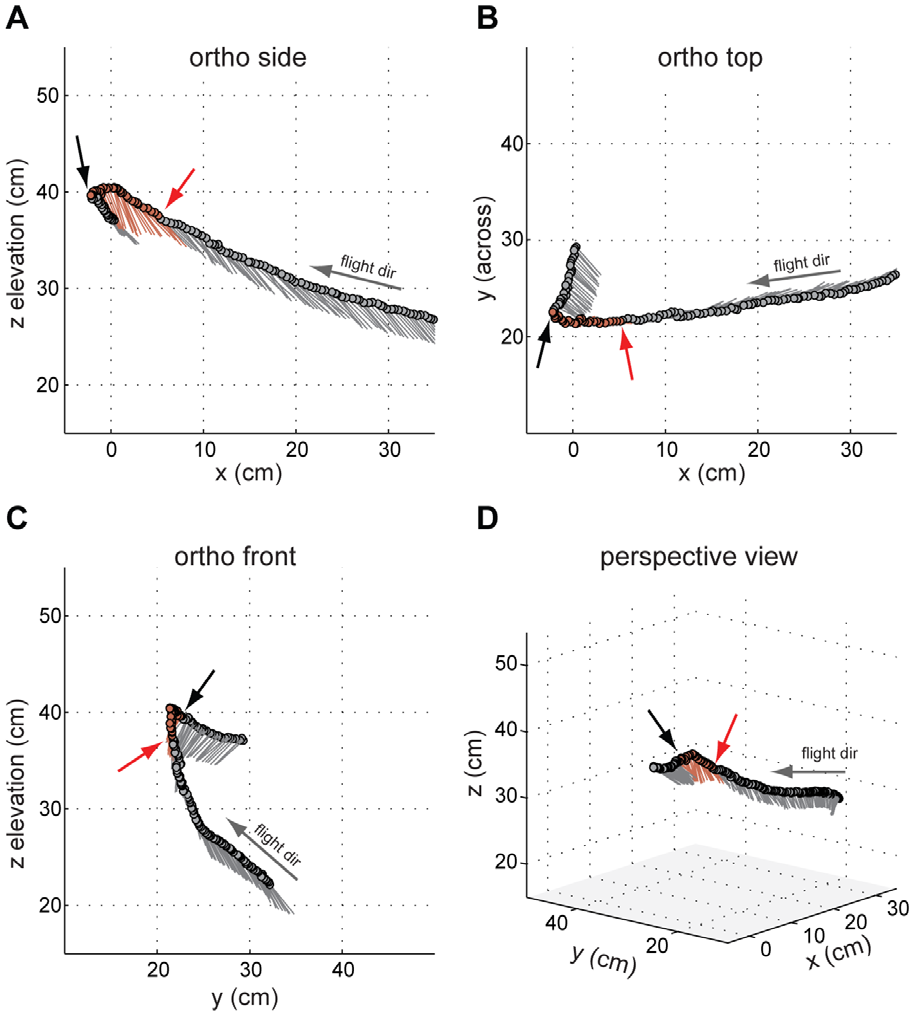
Four projections of a reconstructed flight path while extrinsic muscles of the left antenna were electrically stimulated. Stimulus timing is indicated by the red (onset) and black (end) arrows as well as by red body vector lines connecting the head (circular marker), with the abdominal tip. The arrow labeled “flight dir” indicates the moth's flight direction. (**A**)–(**C**) Orthographic views from the side, top, and front of the wind tunnel, respectively. (**D**) Perspective view (elevation: 15°, azimuth: −50°.)

After repeatedly eliciting antennal deflections in tethered individuals, we performed stimulation experiments in freely flying animals. Although moths were outfitted with electrodes on both antennae, we only succeeded in unilateral stimulation. We successfully recorded video data of in-flight antennal stimulation from six moths (supplemental Movie S2 shows one example). For five moths, we were able to reconstruct 3D coordinates of the body vector (head to abdominal tip) before, during, and after stimulation. As a measure of digitization error, we compared the magnitude of the body vector to the actual body size of a moth. For the representative flight path shown in Figure 3 (see supplemental Movie S2 for the corresponding flight sequence), the body vector length was 4.7 cm (S.D. 0.24 cm) which agrees well with the measured body length of ca. 4.7 cm. The standard deviation of 0.24 cm is an indication of digitization errors, as well as potential changes in the curvature of the moth's body.

**Figure 4:**
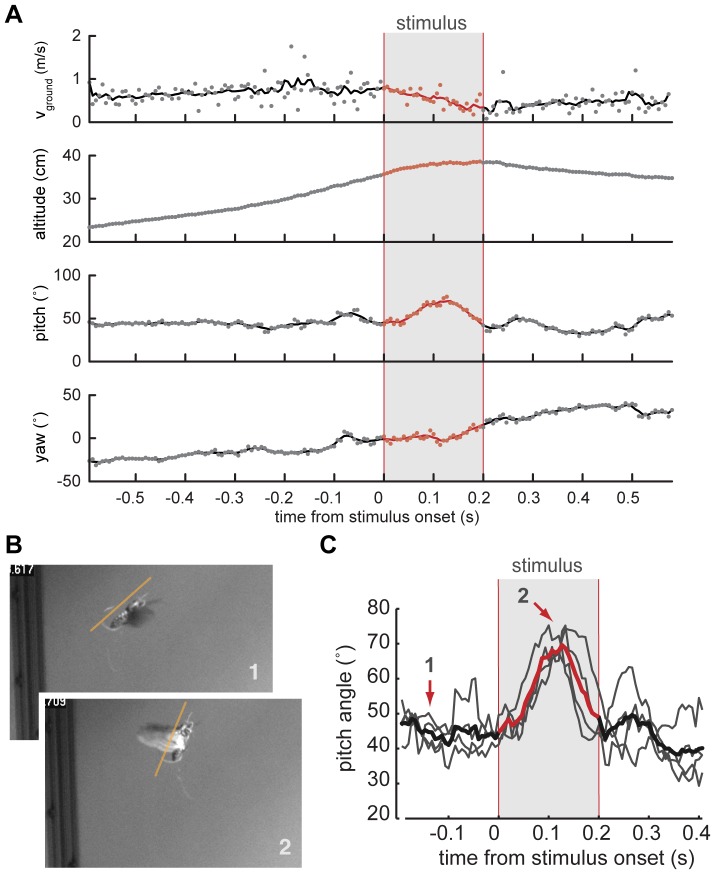
Analysis of changes in flight trajectory elicited by in-flight stimulation of antennal muscles. (**A**) Ground speed (v_ground_), altitude, as well as pitch and yaw heading of a moth's body vector calculated from a 3D reconstruction of a free-flight trial during which extrinsic muscles of the left antenna were stimulated electrically. Stimulus timing is indicated by the gray bar. The change in pitch angle is the only parameter change that could be elicited repeatedly and in a similar fashion in multiple animals. Changes in ground speed, altitude and yaw heading are unique to a specific trial. (**B**) Still images of a moth outfitted with an on-board stimulator shortly before (*1*), and during the stimulus (*2*). (**C**) Average change in pitch angle (red line) for 4 successive trials (underlying grey lines) in one animal shows a consistent response to stimulation. The arrows indicate time points corresponding to the still images in B.

The body vectors showed a distinct increase in their pitch angle shortly after stimulus onset. Additionally, the moth increased its yaw heading, i.e. it turned right, during the stimulus. A better picture of the timing of these events is provided by the graphs in Figure 4, in which the animal's estimated ground speed, its altitude, as well as its pitch and yaw angles are plotted as time increases.

Similar to the representative moth shown in Figure 3, we found that unilateral stimulation of an antenna elicited a pronounced change in pitch angle (ΔTheta>10 degrees) in five of the six animals. Changes in yaw heading, however, occurred less predictably. Table 1 summarizes these changes in pitch and yaw heading elicited by antennal stimulation.

**Table 1:** Summary of responses to free-flight antennal stimulation of extrinsic antennal muscles in six animals.

Animal			Response to stimulation			
animal #	stim. antenna (L/R)	trial	pitch axis (ΔΘ)	yaw axis	Δ_altitude_	Δ_speed_
1	L	1	**+ + +**	n.a.	n.a.	n.a.
		2	**+ +**	**−**	n.r.	**− −**
		3	**+ +**	**+**	**− −**	**− −**
		4	**+ +**	**−/+**	**−**	**− − −**
		5	n.r.	**+**	**−**	n.r.
2	R	1	**+ + +**	**+**	**−**	**+ +**
3	L	1	n.r.	n.r.	**+ +**	n.r.
		2	n.r	n.r.	n.r.	**− −**
4	R	1	**+ +**	n.a.	n.a.	n.a.
5	L	1	**+ +**	**−**	**− −**	**− −**
6	L	1	**+**	**+**	**+ +**	n.r.

Responses in body pitch angle are quantified by calculating the difference ΔΘ between the mean pitch angle (Θ) of a 200 ms period before stimulus onset and the maximum Θ during 400 ms after stimulus onset. All pitch responses were nose-up, with pitch magnitude indicated as follows: **+**: ΔΘ>10°; **+ +**: ΔΘ>15°; **+ + +**: ΔΘ>20° (maximum was 29°). Yaw axis responses are defined as changes in yaw heading that happened within 400 ms of stimulus onset. Yaw responses varied from one stimulation to the next. Animals either turned clockwise (+), counter-clockwise (−), or showed no change (n.r.) in yaw heading. Symbols in the Δ_altitude_ column indicate whether a change in the z-coordinate of the moth occurred after stimulation. A change in altitude of ±10 cm between a 200 ms interval before, and a 200 ms interval after stimulus onset is symbolized with a − or +, depending on whether the moth lost or gained altitude, respectively. Similarly, a change greater than ±15 cm is shown as either − − or ++ (maximum was 22 cm/s ). Similarly, the Δ_speed_ column indicates changes in the mean body speed before and after stimulus onset. (+ +: >1 m/s^2^; −: <−0.5 m/s^2^; − −: <−1 m/s^2^; − − −: <−1.5 m/s^2^(max. was −2.8 m/s^2^). In most cases, stimulation is associated with a decrease in ground speed. In cells labeled “n.a.”, parameters could not be computed because a full 3D path was impossible to reconstruct.

In one animal, we were able to perform 3D - reconstructions of the flight trajectories of 4 successive stimulation events (stimulus train duration:∼230 ms; 34 pulses of 0.25 ms length at 3 V). All stimulus events elicited a transient head-up change in pitch angle. The pitch angle returned to the pre-stimulus value before the stimulus was over, at approximately 200 ms. The maximum of the mean excursion in pitch angle for the four trials was about 24.2° in amplitude (ΔTheta) and occured ca. 127 ms after stimulus onset (Fig. 4c).

## Discussion

Previous studies have clearly demonstrated that antennae in Lepidoptera and other insects fulfill a crucial mechanosensory role that mediates reflexes important for flight control [6]–[8], [14]. In the present study we explored whether indirect stimulation of antenna-based mechanosensors has an effect on the flight trajectory of *Manduca sexta*. We first show that stimulation of extrinsic muscles leads to antennal deflections in resting animals. Such flagellar deflections necessarily stimulate mechanosensory structures within and around the antennal base such as the Böhm's bristles or the scolopedia of the Johnston's organ.

The complex nature of mechanical strain patterns that arise when a moth is flying makes it experimentally extremely difficult to deliver mechanical stimuli that, for example, mimic a natural perturbation. Thus, we had to restrict our stimulus protocol to rather crude antennal deflections that were delivered without any knowledge of their phase within the wing beat cycle. For future experiments, this limitation could be overcome by designing a stimulus circuit that can be triggered on a particular phase in the wing beat signal derived from an on-board accelerometer.

The other limitation of our stimulus stems from the difficulty of targeting extrinsic antennal muscles. In tethered moths, intrinsic muscles located in the first antennal segment (scape) are an easier target for stimulation, and we were able to achieve more controlled deflections of the antennae (data not shown). This approach could unfortunately not be used for free-flight experiments, because when electrodes were placed in the scape the stiffness of the electrode wire prevented moths from positioning their antennae in a natural angle assumed during flight. This slight tension in wires, in turn, prevented them from taking flight. Thus we were unable to map antennal deflection direction to a behavioral response direction.

At the onset of each experiment, the stimulus level was titrated in the resting moth by changing its frequency to elicit antennal deflection in absence of a more generalized response, such as twitching of legs or initiation of flight. We thereby tried to limit the effect of the stimulus to within the local muscles. However, we cannot completely rule out that the stimulus current spread further to produce non-local effects. It is possible that the stimulus current also affected the antennal nerve. That said, the observed changes in flight trajectory are much more likely attributed to excitement of mechanosensory as opposed to olfactory neurons. Thus our results still suggest a role of antennal mechanosensors as a feedback for rapid course corrections.

Using a novel stimulus generator small enough to be carried by a hawkmoth flying in a wind tunnel, we show that unilateral, telemetric antennal stimulation leads to repeatable, transient changes in flight trajectory. More specifically, in-flight antennal deflections in a backwards direction lead to perturbations in the pitch axis of the insect, while any influence on altitude, yaw orientation, or body speed is more varied and depends on other factors, such as the moth's proximity to a wall, or its initial heading with respect to the wind (Table 1, Figure 4.). It is also possible that variation in electrode placement lead to slightly different recruitment of mechanosensors, thus leading to responses in other than the pitch orientation. However, pitch orientation seems to be the most readily disturbed, even though in our experiments antennal stimulation is applied unilaterally.

The pitch angle returned to the pre-stimulus value while the stimulus was still active, at approximately 200 ms. The phasic response might be caused by a stimulus train leading to a transient mechanical stress. By moving the scape, the flagellum will follow this movement with a delay. Stresses experienced by receptors in the pedicel that sits between scape and flagellum are thus phasic. Additionally, the moth's nervous system, presumably via the visual and other mechanoreceptive systems, could react to the perturbation with a compensatory action. The speed of recovery (∼100 ms) would suggest that active compensatory mechanisms play a role in shaping the transient response.

The change in pitch orientation agrees with the effect of the underlying antennal circuit that is involved in abdominal flexion. Antennal mechanoreceptors are used to perceive rotations of a moth around its pitch axis [9]. When rotated without any visual input, a moth responds to this stimulus with a movement of its abdomen. This movement changes the animal's center of mass with respect to its center of lift, and therefore induces torques that can lead to body rotations [9]. The fact that antennal stimulation mostly leads to transient perturbations in pitch is therefore evidence that antennal circuits preferentially stabilize this body axis, as opposed to the roll or yaw orientation.

That the pitch axis might be more important than others for rapid feedback control is also corroborated by computational models that suggest that the body orientation in flying insects is inherently unstable, and that it needs active control to be stabilized [15]–[16]. Pitch rate and attitude, as well as horizontal velocity are three feedback parameters that are sufficient for stabilizing flight in a model of a hovering insect [16]. The relative importance of information about the animal's pitch axis for control could therefore also explain why the effects of responses mediated by antennal mechanosensors are so strongly tied to the moth's pitch orientation.

By augmenting the visual system in signaling changes occurring in the animal's pitch attitude, antennal mechanoreceptors could provide the necessary feedback for stabilizing this inherently unstable axis. This notion agrees well with the observation from the free-flight experiments with cut antenna: the supplemental videos in [8] show moths losing pitch control first when they lack feedback from their antennae. The present results, in which stimulation to antennal mechanoreceptors preferentially leads to pitch instabilities, provide further evidence for a pitch-stabilizing role of insect antennae.

## Supporting Information

Movie S1 **Example of a strong antennal motion elicited by electrical stimulation of extrinsic antennal muscles, filmed at 250 fps.** The tethered moth's head is viewed from above, with an electrode pair implanted in close proximity to the antennal rim of the left antenna. The LED on the left border of the frame indicates the timing of a 3 V square-pulse train of 200 Hz that lasted 200 ms.
(MOV)

Movie S2 **Example of an in-flight stimulation event of antennal muscles in a hawkmoth as seen from three synchronized high-speed cameras filming at 250 fps.** Stimulus timing is indicated by a micro-LED on the stimulator board that is mounted ventrally on the moth's abdomen. In this sequence, stimulation occurs between frames 174 and 223, with the LED best visible in Side Camera 1 (middle panel). This video corresponds to the sequence shown in Figure 3.
(MOV)
